# Online scientific research on placentophagy: a bibliometric analysis

**DOI:** 10.61622/rbgo/2024AR04

**Published:** 2024-03-15

**Authors:** Paloma Elisama de Oliveira Morais, Melissa Santos Nassif, Andreia Cristina Barbosa Costa, Patrícia Scotini Freitas, Rômulo Severo Sampaio, Isabelle Cristinne Pinto Costa

**Affiliations:** 1 Universidade Federal de Alfenas Alfenas MG Brazil Universidade Federal de Alfenas, Alfenas, MG, Brazil.; 2 Universidade Federal da Paraíba João Pessoa PB Brazil Universidade Federal da Paraíba, João Pessoa, PB, Brazil.

**Keywords:** Placenta, Pregnancy, Postpartum period, Alternative therapies, Bibliometric indicators

## Abstract

**Objective:**

To classify the bibliometric indicators of online scientific research on placentophagy.

**Methods:**

A bibliometric study was conducted to quantify the scientific production of authors and institutions with the aim of highlighting the growth and impact of these publications nationally and internationally. The Bradford Law, network maps, and textual statistics were used, with searches conducted in libraries and databases in October 2021.

**Results:**

The sample consisted of 64 articles, whose primary authors were associated with 49 institutions, and mostly with degrees in anthropology. The United States of America was the country that published the most papers on the theme, and most studies were reviews with individual production. Through the term analysis, it was found that the predominant themes regarding placentophagy were the following: Alternative therapy for women's health, methodologies used for research in this area, period of placenta ingestion (postpartum period), and its benefits.

**Conclusion:**

The bibliometric indicators found are essential for the development of future research.

## Introduction

The placenta is an organ shared between mother and fetus, undergoing significant anatomical changes and constant adaptation to the uterine environment throughout pregnancy, which is vital in the maternal-fetal transfer of gases and nutrients.^(1,2)^

However, after delivery it no longer serves any purpose and therefore a disposal method must be found. Some women place sentimental value on the organ, which provides nutrition, circulation, and maintenance for their child during pregnancy.^(3,4)^ Therefore, through beliefs, traditions, or previous birth experiences, the destination of the placenta for these women is surrounded by rituals as a form of appreciation and admiration for the organ. Among these, placentophagy can be mentioned.^(3)^

Placentophagy consists of consuming the placenta in the postpartum period, which can be eaten raw, after being heated, dried, and powdered, or encapsulated as a dry powder.^(5)^ This act is becoming increasingly common, especially in high-income countries.^(3,6)^

Despite the apparent interest in the United States of America, there are currently no scientifically validated benefits of human placentophagy.^(7)^ Nonetheless, proponents of placentophagy claim various positive effects, including the prevention of postpartum depression, general improvement in mood and energy, enhanced milk supply, availability of important micronutrients, and reduction in postpartum bleeding.^(8,9)^ Some of these benefits, as observed in animal contexts, can be attributed to the elevated content of estrogen and lactogen present in the placenta. However, it is noteworthy that when drawing parallels with animal behaviors, it becomes imperative to clarify that the primary justification for this association does not reside in the intention to evade detection by predators, a trait not applicable to the human realm. Despite these assertions, it is valid to highlight that such conclusions predominantly retain a theoretical nature, lacking robust scientific evidence for support. Up to the present moment, the evidence corroborating the beneficial effects of placentophagy in humans remains confined to studies based on self-reports and anecdotal accounts.^(7)^

Therefore, the need to develop a bibliometric study on human placentophagy emerges, since publications in the national and international literature are incipient, in addition to the gap in knowledge regarding the theme and the entire process of orientation that permeates it among health professionals. Thus, the contextual analysis of scientific research will provide the necessary knowledge to conduct the clinical practices performed by these professionals.

Furthermore, it aims to minimize the gap in this field of scientific knowledge regarding the nature of the scientific production on human placentophagy, contributing to the scientific community that has as interest in studying this practice, since it will present the distribution of production by year, geographic region, identify the journals that are most dedicated to the subject, and the most productive authors, among other aspects.

In light of the above, this study aims to classify the bibliometric indicators of the scientific production available online that address human placentophagy.

## Methods

This is a descriptive bibliometric study with a quantitative documentary-based approach, which is a growing format in the health area, and is used to quantify the authors’ and institutions’ production and productivity, aiming to highlight the scientific growth and impact of these publications in the international context.^(10)^

According to the methodological description, data collection was performed in a single step, on October 17th, 2021. Initially, a preliminary search was conducted in the PubMed database to determine the most commonly used keywords and controlled descriptors for indexing studies related to human placentophagy. After that, a librarian assisted in choosing the best search strategies that would generate the most information in the databases and libraries used.

The controlled vocabulary was selected from the Descriptors in Health Sciences: "*Placenta*"; "*Saúde da Mulher*" and "*Período Pós-Parto*", and from the Medical Subject Heading: "Placenta"; "Women's Health" and "Postpartum Period". Furthermore, to achieve a targeted search strategy, the following keywords were also used: "*Placentofagia*"; "Placentas"; "Placentophagia"; "Placentophagy"; "Ingesting placenta"; "Eating afterbirth"; "Placenta consumption"; "Eating placenta"; "Women Health"; "Woman's Health"; "Postpartum"; "Postpartum Women", and "Puerperium", combined with the Boolean operators AND and OR.

The search strategy used (Chart 1) was to the specific needs of each library or database to be searched for this review, namely: Web of Science, Scopus, Cochrane Library, PubMed, Cumulative Index to Nursing and Allied Health Literature (CINAHL), Biomedical Answers (Embase), Latin American and Caribbean Health Sciences Literature (LILACS), and to identify unpublished studies on the topic, a gray literature search was conducted on Google Scholar.

**Chart 1:** Search strategies used according to the database/library selected

Database/Library	Search strategy
Web of Science	(Placenta) AND (Postpartum Period)
Scopus PubMed CINAHL	(Placenta) AND (Women's Health) AND (Postpartum Period)
Cochrane Library	(Placenta) OR (Placentophagy) AND (Women's Health) AND (Postpartum Period)
Embase	(Placenta) AND (Women's Health) AND (Postpartum)
LILACS	(*Placenta*) AND (*Período pós-parto*)

The inclusion criteria were the following: primary or secondary studies that address human placentophagy, regardless of the design type. No limitations were set regarding the publication year and language of the articles. Letters, commentaries, editorials, and expert opinion articles were excluded, as they would not be suitable to meet the review objectives. The flow chart proposed by the Preferred Reporting Items for Systematic Reviews and Meta-Analyses (PRISMA) was used for the screening and selection process of the studies.^(11)^ After running the database search, the retrieved articles were exported to EndNote, and duplicates were discarded (https://clarivate.com/webofsciencegroup/support/endnote-online/). Subsequently, the articles were exported to the Rayyan (https://www.rayyan.ai/) application, which is the software used for the selection step. Therefore, the titles and abstracts of the articles were read, following the eligibility criteria. Those that were selected were read in full aiming to exclude those that failed to meet the study's criteria and guiding question. It is noteworthy that the selection of articles was carried out by two independent reviewers and, in cases of divergent decisions, these were settled by a third reviewer. Aiming to improve data organization and analysis, a table was built using Microsoft Excel, considering the following bibliometric indicators: Country; most productive institutions; main authors’ training areas; language; the number of authors; co-editing authors with the greatest production on human placentophagy; research participant population; publication modality; environment studied; dispersion of the journals in productivity zones, and descriptors/keywords. The data obtained were grouped and analyzed using descriptive statistics (frequency and percentage).

Bradford's Law was used to analyze the individual productivity of the scientific journals, as it enables the division into zones of the total number of journals found. In this sense, the journals were divided into 4 zones. Zone 1 (core) is composed of the journals with the highest productivity (containing the first quarter of the total review articles), followed by zone 2, with the next 25% of the total number of journals. The third and fourth zones consist of journals that published articles only a few times on the topic.^(12,13)^

For this Law, data tabulation was performed with subsequent application of the analytical formula Bm = (1.781 x Ym) 1/p to check the result. It is considered that Bradford multiplier (Bm) is a constant; "Ym" is maximum productivity, and "p", is the number of zones.^(14)^

The data analysis allowed the results to be presented descriptively and with the aid of figures, charts, and tables. The generation of the network maps was carried out with the support of the VOSviewer software version 1.6.14, which enabled the research data in the databases to be exported and then processed, and the assessments to be drawn up. Such maps use loops and colors to emphasize authors or concepts that are interconnected, and the following analyses were performed: Keyword relationships and co-citation among authors.

## Results

**Figure 1:**
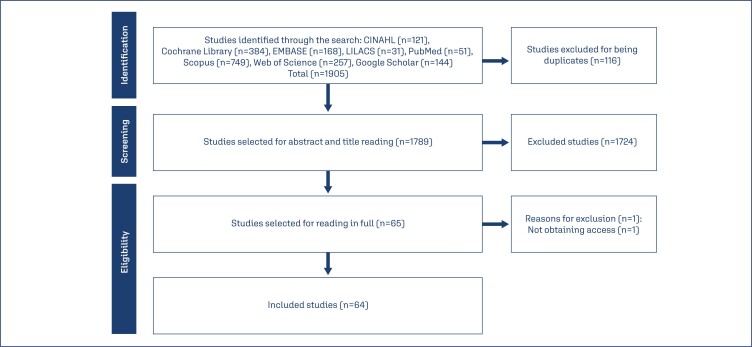
Study identification and inclusion process

A total of 1905 articles were identified in the first search, and after excluding 116 duplicates, a total of 1789 studies remained. After the title and abstract analysis, and the application of the eligibility criteria, 65 articles were pre-selected for reading in full. Of these, 64 were included in this review's final sample. The search and selection path followed the PRISMA 2020 Flow Diagram guidelines, which are shown in figure 1.

### Country, most productive institutions, and training areas of the main authors

Table 1 presents data related to the first authors of the publications.

**Table 1:** Results related to the country, affiliation, and training area of the main authors

Variables		n(%)
Main author's country		
	United States of America (USA)	32(50,00)
	Germany	6(9,38)
	Canada	4(6,25)
	Australia, The United Kingdom, Turkey and Russia	2(3,13)*
	Argentina, Austria, Belgium, Chile, Colombia, Czech Republic, England, Malaysia, Mexico, Nigeria, Peru, Poland, Slovenia, Spain	1(1,56)*
First author's institution		
	University of Nevada	10(15,63)
	Jena University Hospital (Germany)	3(4,69)
	Not stated	3(4,69)
	Remark: 1 institution with 2 articles	2(3,12)
	Remark: 46 institutions with 1 article each	46(71,87)
Training area (main author)		
	Not stated	14(21,87)
	Anthropology	11(17,19)
	Medicine	9(14,07)
	Student in the area of healthcare	6(9,38)
	Obstetrics	6(9,38)
	Nursing	4(6,25)
	Psychology	4(6,25)
	Law	2(3,12)
	Biological Sciences, Conference, Lactation Consultant, Industrial Engineering, Philosophy, Medical Genetics, Neuropsychology, Pediatrics	1(1,56)†

* Value of each country;

† Value of each training area

### Languages available for reading in full

The prevailing language was English, in 57 (89.06%) publications, followed by two (3.13%) articles in Spanish, and two (3.13%) in Turkish. In addition, there was one (1.56%) article in Polish, one (1.56%) in Russian, and one (1.56%) in Slovene.

### The number of authors by article

Individual production was present in 24 (37.5%) publications, followed by 10 (15.62%) articles with three authors, and 10 (15.62%) articles with six authors. The proportion of two authors by each article was present in six (9.38%) studies, and the proportion of four and five authors both presented five (7.81%) articles each. Besides these, two (3.13%) of the articles had 10 authors, one (1.56%) article had seven authors, and one (1.56%) had eight authors.

### Co-citation of the authors with the most published research on placentophagy

**Figure 2:**
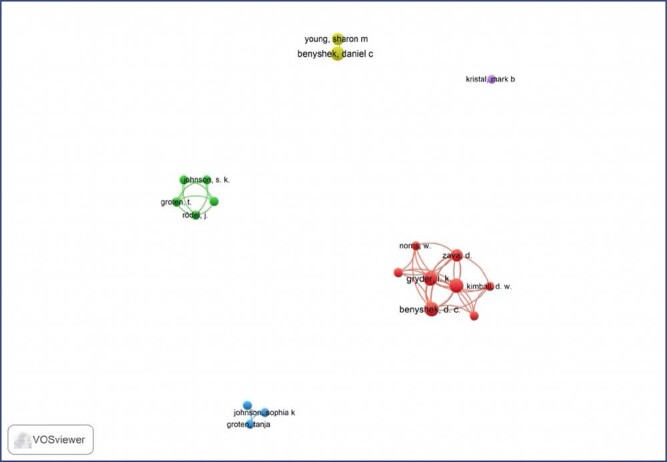
Co-citation network map among the main authors of the studies included in this study

**Figure 3:**
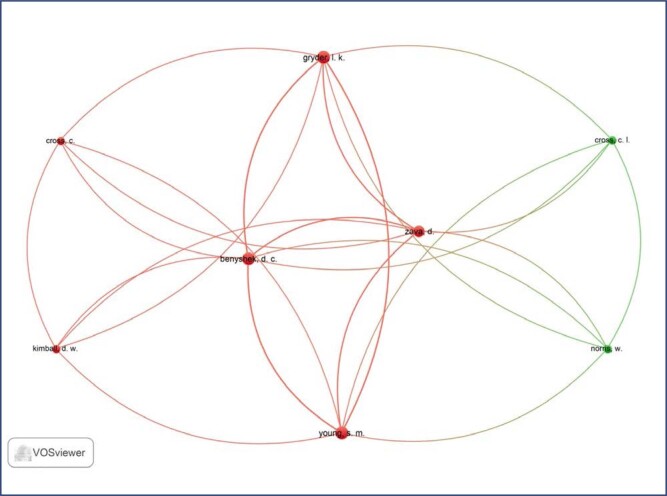
Network map of co-citation among the main authors of the studies included in this study

Figures 2 and 3 present the co-citation network produced based on the references belonging to the main authors. As authors are listed together, a link is formed, inferring a connection or relationship among them. The size of the bubble indicates the normalized number of citations received by the articles, and the thickness of the lines represents the strength of the co-citation links. The color of the bubble indicates the cluster with which the author is associated, which represents a set of included authors. Figure 2 shows a total of 19 authors, out of 176, who presented at least two co-citations. Then, for each of the 19 authors, the total strength of the co-citation links with other authors was calculated. It is noteworthy that some of the 19 items in the network are not connected among themselves.

Figure 3 presents the authors who are connected. The largest set of items connected consisted of eight items, noting that the network map generated in this study presents a total of two clusters. The identification of the most cited authors occurs by using a cutoff number established by the VOSviewer Software, thus, the authors who stand out in the red cluster on the topic of placentophagy are the following: Benyshek, D. C., Cross, C., Gryder, I. K., Kimball, D. W., Young, S. M., and Zava, D. Meanwhile, in the green cluster, the authors who stand out are Cross, C. I., and Norris, W.

### Publication modality, studied population, and development environment

It is worth noting that of the 64 studies identified, 48 were evaluated regarding the population, considering that 16 were review studies. Most of the research subjects were pregnant women (n=7; 14.6%) and placenta samples (n=6; 12.5%). In addition, it was found that four (8.33%) articles had puerperae as participants, four (8.33%) had the general population in order to identify their opinion regarding this practice, three (6.25%) were carried out with parents and obstetricians, two (4.17%) were carried out with children whose mothers performed placentophagy, one (2.08%) was carried out with obstetricians and puerperae, one (2.08%) with pregnant women and puerperae, one (2.08%) with women suffering from psychiatric disorders, one (2.08%) with healthcare professionals and women, and one (2.08%) with pregnant women, puerperae, and specialists in placenta encapsulation. It is noteworthy that in 17 (35.42%) studies there was no population described.

Regarding methodology, 16 (25%) studies were literature reviews and four (25%) of these had been described as systematic. In addition, eight (12.5%) articles provided information, six (9.38%) were randomized, double-blind, placebo-controlled pilot studies, two (3.13%) were case reports, two (3.13%) were descriptive studies, two (3.13%) proposed hypotheses, one (1.56%) was a cross-sectional survey, one (1.56%) was a thematic analysis, one (1.56%) was a cross-cultural ethnographic search, one (1.56%) was an observational, longitudinal prospective, and sequential nutritional study of cases and controls, one (1.56%) study had mixed methods (cross-sectional research and online discussions), one (1.56%) was a qualitative study, one (1.56%) had an interpretative qualitative approach, one (1.56%) was a limited systematic research, one (1.56%) was an analytic research, one (1.56%) was a quantitative study of simple descriptive, cross-sectional, prospective design, one (1.56%) was a personal history, one (1.56%) was a study with a qualitative approach with an exploratory research design, one (1.56%) explored the legal implications of placentophagy, and one (1.56%) was a double-blind placebo-controlled article. It is also noteworthy that 14 (21.88%) studies have no described methodology.

Regarding the location where the studies were carried out, 18 (28.12%) did not describe it. The others were conducted in obstetrics departments (n=15; 23.44%), academic hospitals (n=5; 7.81%), public hospitals (n=4; 6.25%), universities (n=3; 4.69%), outpatient clinics (n=2; 3.13%), private clinics (n=2; 3.13%), and in a refugee camp (n=1; 1.56%). Other forms of research development were carried out through dissemination on social media, with an incidence of 21.87% (n=14) of the total study locations.

### Journal allocation in productivity zones

Given the 64 studies included, it is noteworthy that six (9.38%) were disregarded during the application of Bradford's Law, as these studies are not bound to any journals. These were published in libraries and archives of the universities associated with the authors. Applying Bradford's Law, a total of 58 articles were distributed in 45 journals. Considering that 25% of the 58 articles total represents 14.5 articles and that there is no possibility of considering only part of the articles of a journal, a mathematical approximation was used aiming to encompass all the articles of each journal, which resulted in 15 articles for zone 1, so that such calculation was based on the Bm obtained for the considered data series. The Bm value according to the calculation in analytical form was Bm≈1.63. The first six journals within zone 1, which consists of approximately 25% (n=15) of the 58 articles are the following: Ecology of Food and Nutrition (n=4; 6.90%), Journal of Midwifery and Women's Health (n=3; 5.17%), American Journal of Obstetrics & Gynecology (n=2; 3.45%), American Journal of Physical Anthropology (n=2; 3.45%), Journal of Obstetric Gynecologic and Neonatal Nursing (n=2; 3.45%), and Placenta (n=2; 3.45%). Zone 2 consists of five journals, which together have published a total of nine articles. Zone 3 consists of 16 journals that have published only one article each, totaling 16 articles. Finally, zone 4 consists of 18 journals, which together have published a total of 18 articles.

### Descriptor/keyword analysis

With the aim of exploring the themes addressed, an analysis of the frequency of descriptors/keywords of the studies included in the sample was performed, which enabled the validation of the main lines of study. It is noteworthy that the VOSviewer Software analyzes a relevance score of these terms and those which are the most relevant are selected. Therefore, the number of terms selected was 28.

**Figure 4:**
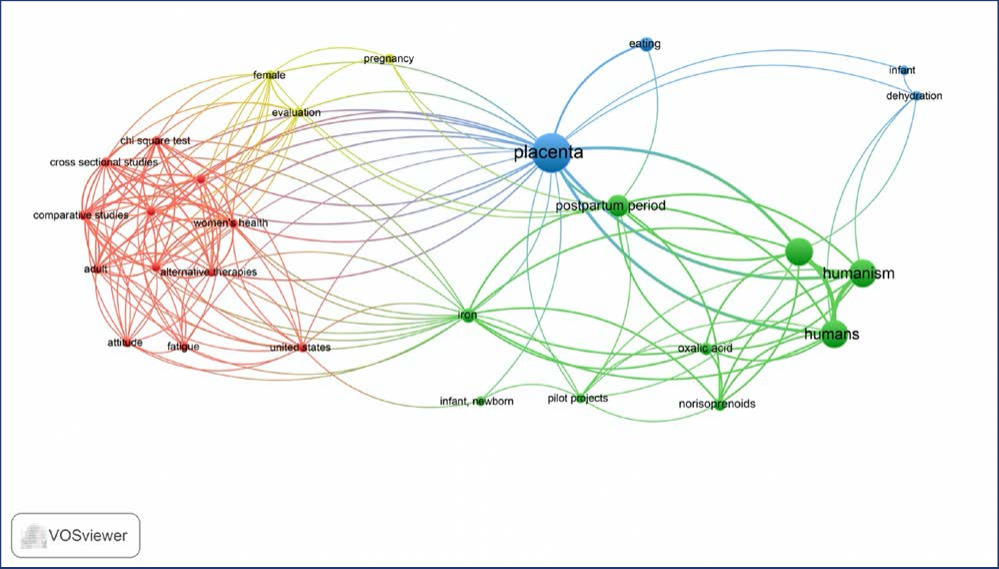
Co-occurrence network map of descriptors/keywords related to placentophagy in the year 2022 (January/February)

Figure 4 reveals the existence of four clusters related to placentophagy. The red cluster identifies themes related to the development of research on the subject, indicating that this alternative therapy (*alternative therapies*) is more often investigated in humans (*human*) in the adult phase (*adult*), in the area of women's health (*women's health*), especially in the United States (*United States*), aiming to verify a relationship with fatigue (*fatigue*) and the women's behavior (*attitude*) of ingesting their placentas after birth, using the following resources for conducting research: The Chi-squared test (*chi square test*), comparative studies (*comparative studies*), cross-sectional studies (*cross sectional studies*), data analysis software (*data analysis software*), and descriptive statistics (*descriptive statistics*).

The green cluster indicates that the ingestion of the placenta (*placenta*) in humans (*humans*) occurs in the postpartum period (*postpartum period*) to improve iron (*iron*) and oxalic acid levels (*oxalic acid*) in infants and newborns (*infant, newborn*), according to pilot projects (*pilot projects*). The blue cluster indicates that the act of eating (*eating*) the placenta (*placenta*) improves the infant's dehydration (*infant*, *dehydration*) and, finally, the yellow cluster indicates that the placenta is an organ present throughout the entire pregnancy evolution (*pregnancy*, *evolution*) of women (*female*).

## Discussion

Although the literature on placentophagy has recently grown, this review found a scarcity of studies scientifically proving its benefits and harms to the health of those involved. It was observed that the practice is performed by postpartum mothers who seek to improve their health or as a ritual of gratitude to the organ that nourished their child during pregnancy. Additionally, women also seek to increase their milk production to improve their infant's nutrition.

This bibliometric review found that the largest scientific production stems from the USA, which may be justified as it was one of the first countries where placentophagy was practiced. In the mid-1980s, when isolated studies were published regarding its supposed benefits, the practice emerged and became increasingly known in this country. It is pertinent to mention that approximately one-third of USA postpartum women in outpatient settings consume their placenta, and women who have had home deliveries are more likely to engage in placentophagy.^(15)^

The most productive institution, the University of Nevada, offers nursing, medicine, and public health courses, focused on training professionals dedicated to providing care to the population, teaching, and research. In addition, it has the Laboratory of Nutrition and Reproduction, which has human placentophagy as one of its research areas.^(16)^

Regarding the main author's training, it was found that anthropology professionals were prevalent. This finding is justified by the need to provide further cultural and historical discussions on the subject, as there are indications that placentophagy may have been practiced in the past.^(5)^ In contrast, this review found only four studies with nurses as the main authors, which is surprising, since these professionals are entirely related to the care of women during childbirth and puerperium, and from the moment they become aware of this practice through research, they can discuss the best options with the families.

Regarding language, it was found that most studies are written in English, indicating the authors’ concern with communicating their data to the greatest number of people through a more widely understood language. In addition, articles in English are cited more often than those in other languages, which means more prominence and reliability.^(17)^ It is worth noting that this finding is consistent with several bibliometric studies.^(18,19)^

Regarding the number of authors by article, contrary to what the literature recommends, individual production prevailed. However, it is noteworthy that collaboration among authors benefits the institutions and countries which they are associated with, as well as the entire scientific community, by enabling shared scientific knowledge in addition to specializing and furthering research.^(20)^

The authors who stand out on this theme are Benyshek, D. C., Gryder, L. K., and Young, S.M., who were the most prevalent, being cited five times by the studies in the sample, each. The accuracy of this data can be determined by viewing the largest bubbles on the network map, which indicate the number of citations received by the articles. All three researchers have a background in anthropology and belong to the Department of Anthropology at the University of Nevada, with the first one specializing in human placentophagy. In addition, Benyshek is a current member of the Nutrition and Reproduction Laboratory at this university, and the other two are former participants.

Most of the population participating in the included studies were pregnant women. According to a study, of 23,242 women who planned births with a midwife in the United States, 30.8% consumed their own placenta.^(15)^ This data shows that, even with limited scientific and corroborative information regarding its benefits, women have been trying to learn about and practice placentophagy.^(21)^

Regarding methodology, it is noteworthy that 14 studies in the sample had no described methodology. These data show that the level of scientific evidence of research related to placentophagy is generally weak, since in most articles no description of the methodology is provided, and the information is fragile. Therefore, studies that used a methodological approach that would provide high levels of scientific evidence are incipient. Especially for the health area, the scientific basis is extremely important at the moment of decision-making.^(22)^

Regarding the location where the studies were carried out, they were conducted in obstetrics departments, hospitals, clinics, universities, and outpatient clinics, a result previously expected since they were carried out primarily with pregnant women.

Through the application of Bradford's Law, it was possible to verify the existence of a small cluster of journals that portrays the theme more broadly, and an extensive peripheral region divided into zones, where the increase of journals that decrease the production of studies published on placentophagy is noted. In this cluster, some journals that aim to potentially transform women's health in their clinical practice and promote an impact on the knowledge of health and disease stand out, and may present a tendency to establish a core of supposedly superior quality and greater relevance in this field of knowledge.

Regarding the investigation of descriptors and keywords, it was possible to note that research on placentophagy covers the following predominance of themes: Alternative therapy aimed at women's health, methodologies employed for research in this area, period of placenta ingestion (postpartum period), and the benefits of this practice.

Regarding placentophagy as an alternative practice, it is considered as such, since it is not a medication practice. According to a study, women with a higher socioeconomic profile and at least one undergraduate degree practice placentophagy more often than low-income women, and this may be due to the fact that these practices are not always covered by health insurance, especially outside of Brazil.^(23)^

Of the women who choose to ingest their placentas, 73.1% seek prevention of postpartum depression and 14% seek nutritional and iron supplementation after their pregnancy ends.^(17)^ However, small differences were identified in the mood of women who practiced placentophagy when compared to women in the placebo group in a study by Young et al.^(24)^ In addition, some studies have proven that encapsulated placenta may provide only a modest source of some micronutrients to women, and fail to provide the recommended minimum daily iron intake.^(25,26)^

Furthermore, according to a study, women who practice placentophagy also seek to improve the quality and quantity of their milk.^(8)^ This benefit was studied in 1917, and it was reported that a change in the chemical composition of milk occurs, including an increase in prolactin, in women who ingest their dehydrated placenta.^(27)^ However, in 2019 another study contested this result, proving that the practice does not affect postpartum prolactin levels.^(28)^

Regarding the disadvantages, a late infection of an infant by group B *Streptococcus agalactiae* (GBS) was reported after the mother used an encapsulated placenta.^(29)^ Another study has indicated that when the placenta undergoes thermal processing, this process considerably reduces the colony forming units of GBS and *E. coli*, rendering clinical infection unlikely to occur through this practice.^(21)^

Several forms of placenta consumption are possible, such as raw, cooked, roasted, dehydrated, encapsulated, or in smoothies and tinctures,^(8)^ with the most common technique being encapsulation after steam cooking and dehydration.^(7)^ Companies like Placenta Benefits (PBi) Ltd offer the service of preparing placenta for consumption, charging between $200 and $400 for encapsulation.^(9)^ Advocates of the technique claim that it preserves all the benefits that the placenta can offer, but there is data indicating that some nutrients, such as iron, may be lost during the process and that the ingested amount may not have biological activity or clinical benefit.^(30)^ Due to this lack of scientific evidence, healthcare professionals are not responsible for offering placentophagy to their patients, and it is their responsibility to provide directive counseling recommending against the consumption of placenta.^(7)^

As a limitation of this study, the low number of randomized clinical trials can be mentioned, which hinders the generalization of the results. In addition, a low number of systematic reviews is available, which are classified as level I in the evidence level pyramid, making it difficult to reach results that can be implemented in clinical practice.

## Conclusion

The bibliometric indicators investigated show that most scientific research on placentophagy stems from the University of Nevada in the USA, with a production system of individual studies available in English, which are review studies, developed by anthropology professionals, and including mostly pregnant women assisted by obstetrics departments. Through the relationship maps, it was possible to evidence the emergence of co-citation clusters and the most used descriptors/keywords. Considering the health sector, it is expected that these findings may spark the interest of researchers to develop studies that address this theme, since it is a growing practice in humans, but knowledge of its benefits and hazards is limited, as well as how the placenta should be prepared for this purpose. Moreover, monitoring the development of international academic research in this area is of utmost importance to guide the future of research related to the subject.
